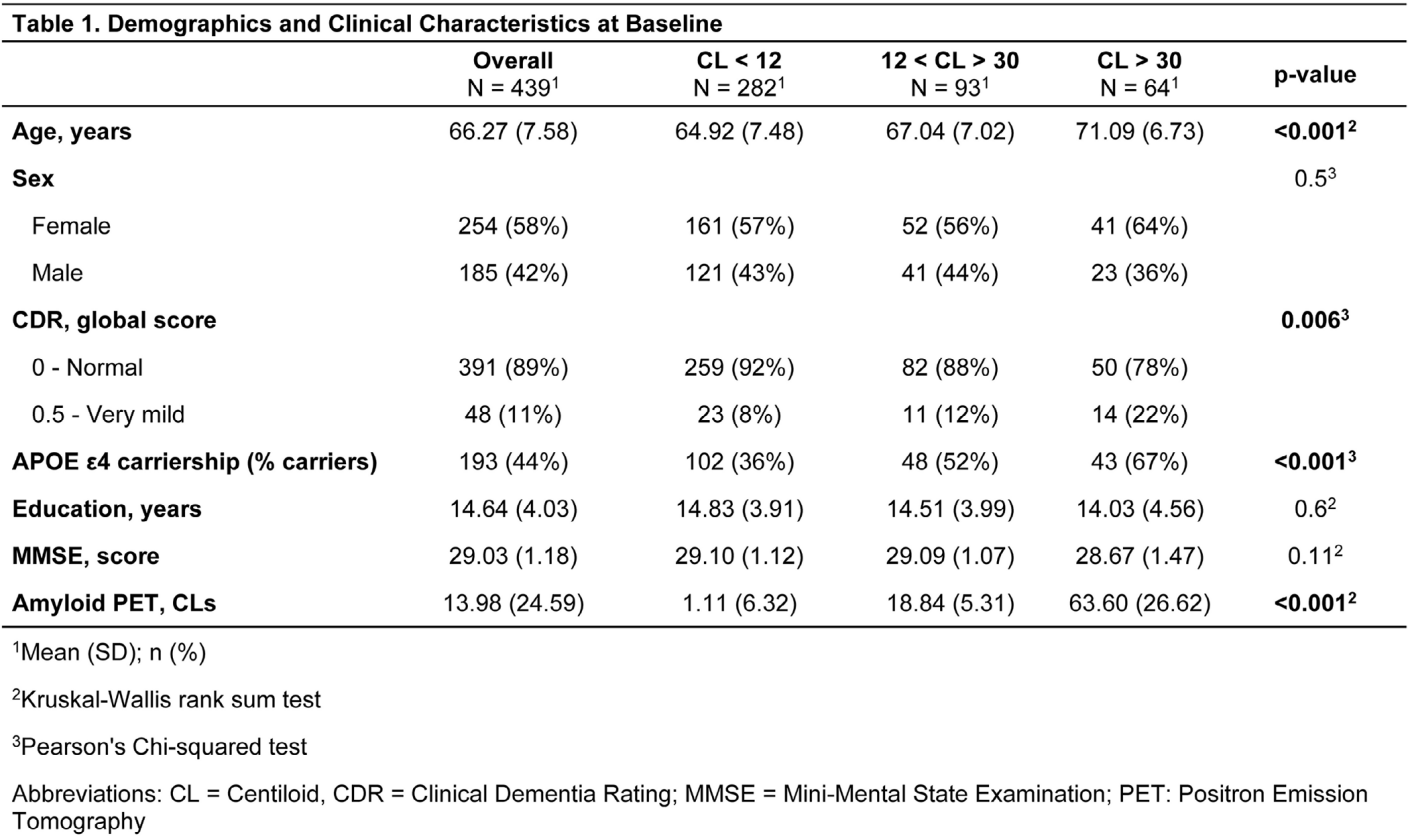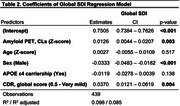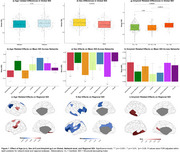# Brain structural‐functional coupling is differentially affected by age, sex, and amyloid burden in older individuals without dementia

**DOI:** 10.1002/alz70862_109902

**Published:** 2025-12-23

**Authors:** Prithvi Arunachalam, Francesca Treves, Leonard Pieperhoff, Luigi Lorenzini, Mario Tranfa, Federico Masserini, Maria G. Preti, Giuseppe Pontillo, Lyduine E. Collij, Tommy A. A. Broeders, Menno M. Schoonheim, Linda Douw, Craig Ritchie, Mercè Boada, Marta Marquié, Pieter Jelle Visser, Juan Domingo Gispert, James H. Cole, Frederik Barkhof, Alle Meije Wink

**Affiliations:** ^1^ Amsterdam University Medical Center (Amsterdam UMC), Amsterdam, North Holland Netherlands; ^2^ University of Pavia, Pavia Italy; ^3^ University of Naples Federico II, Naples Italy; ^4^ University of Milan, Milan Italy; ^5^ Ecole Polytechnique Fédérale de Lausanne (EPFL), Lausanne Switzerland; ^6^ University of Geneva, Geneva Switzerland; ^7^ University College London (UCL), London UK; ^8^ Lund University, Lund Sweden; ^9^ Scottish Brain Sciences, Edinburgh, Scotland UK; ^10^ Ace Alzheimer Center Barcelona – International University of Catalunya (UIC), Barcelona Spain; ^11^ Maastricht University, Maastricht Netherlands; ^12^ Karolinska Institutet, Stockholm Sweden; ^13^ Barcelonaβeta Brain Research Center (BBRC), Pasqual Maragall Foundation, Barcelona Spain

## Abstract

**Background:**

Brain function emerges from structural pathways enabling neural connectivity and communication. However, the extent of structural‐functional coupling varies across brain regions, and its alteration in aging and early Alzheimer’s disease (AD) remains unclear. This study investigated variations in coupling associated with age, sex, and amyloid burden in a cohort of older adults without dementia.

**Method:**

We included 439 participants from the AMYPAD consortium with diffusion‐weighted imaging (DWI), functional magnetic resonance imaging (fMRI), and amyloid positron emission tomography (PET) available. Structural and functional connectomes comprising 100 nodes from the Schaefer atlas were built using QSIprep v0.19.0 and fMRIPrep v23.0.1. Global cortical amyloid burden was assessed using the Centiloid scale. Structural‐Decoupling Index (SDI) was computed using a graph signal processing framework, which projects functional data onto the structural connectome, filtering it into coupled and decoupled components. The ratio of these components yields regional SDI values, reflecting the degree of decoupling between brain structure and function, with lower SDI indicating higher coupling and vice versa (https://www.github.com/gpreti/GSP_StructuralDecouplingIndex). Linear models were used to investigate the effects of age, sex, and baseline amyloid burden on global, network‐level, and regional SDI, correcting for *APOE* ε4 carriership and global Clinical Dementia Rating score.

**Result:**

Cohort characteristics are summarized in Table‐1. Age had no association with global SDI (Table‐2) but was positively associated with SDI in the somatomotor network (Figure‐1b). Regionally, higher SDI was found in somatomotor and dorsal attention regions, while sparse decreases were observed in the default mode and frontoparietal network regions (Figure‐1c). Males exhibited lower SDI at both global (Table‐2) and network‐level (Figure‐1e). Regionally, males demonstrated lower SDI mostly in fronto‐temporal regions (Figure‐1f). Higher amyloid burden was associated with higher SDI at the global (Table‐2; Figure‐1g) and network‐levels, including the default mode, frontoparietal, ventral attention, and visual networks (Figure‐1h). Regionally, global amyloid burden was linked to higher SDI in the inferior temporal regions (Figure‐1i).

**Conclusion:**

Our findings demonstrate that age, sex, and global amyloid burden independently and differentially relate to structural‐functional coupling across brain scales. This work highlights the sensitivity of multimodal brain network analyses in detecting changes that may reflect distinct pathophysiological processes in aging and early AD.